# Asystole Likely Due to a Vagal Reflex During Routine Gynecologic Surgery: A Case Report

**DOI:** 10.7759/cureus.115281

**Published:** 2026-08-27

**Authors:** Max Lebowitz, Tiffany Azzarello

**Affiliations:** 1 Medicine, St. George's University, True Blue, GRD; 2 Obstetrics and Gynecology, St. Vincent's Medical Center, Toledo, USA

**Keywords:** asystole, cardiac arrest, cardiopulmonary resuscitation, gynecologic surgery, intraoperative bradycardia, laparoscopic surgery, peritoneal insufflation, pneumoperitoneum, vagal reflex

## Abstract

Vagal-mediated cardiovascular responses can occur following carbon dioxide pneumoperitoneum during laparoscopic surgery. In rare cases, an exaggerated vagal reflex may result in profound bradycardia or asystole.

We report the case of a 44-year-old woman (American Society of Anesthesiologists physical status II) who developed abrupt bradycardia progressing to asystolic cardiac arrest immediately after abdominal insufflation during an elective minimally invasive gynecologic procedure. The planned operation included hysteroscopy with MyoSure (Hologic, Inc., Marlborough, MA, US) resection, NovaSure (Hologic) endometrial ablation, Acessa (Hologic) radiofrequency fibroid ablation, diagnostic laparoscopy, and salpingectomy for symptomatic uterine fibroids refractory to conservative management. Her past medical history was notable only for uterine fibroids and recently diagnosed hypertension. Preoperative laboratory testing and vital signs were within normal limits, and electrocardiography demonstrated normal sinus rhythm without clinically significant rhythm abnormalities.

Immediately following the bradycardia, one milligram of atropine was administered intravenously. The patient then became asystolic, pneumoperitoneum was evacuated, and cardiopulmonary resuscitation was initiated. Return of spontaneous circulation was achieved after a single round of compressions, with restoration of normal sinus rhythm. The intra-abdominal portion of the procedure was subsequently aborted, and the patient remained hemodynamically stable postoperatively.

At cardiology follow-up nine days postoperatively, the patient reported prior vasovagal-type episodes, including syncope following influenza vaccination and near-syncope during magnetic resonance imaging with intravenous contrast.

This case highlights the importance of rapid recognition and management of vagally mediated cardiac events in the operating room. It also raises the question of whether targeted preoperative assessment for prior syncope or vasovagal episodes may help identify patients at increased risk for exaggerated vagal responses during procedures involving pneumoperitoneum, a potential area for future investigation.

## Introduction

Laparoscopic surgery has become the preferred approach for many abdominal and gynecological procedures. It generally results in less trauma to abdominal wall tissue, reduced time to heal, and a faster discharge. It is also associated with decreased wound infections and lower perioperative morbidity [1]. Although it has transformed modern surgical practice, laparoscopy is not without unique physiologic challenges. While the required creation of pneumoperitoneum commonly induces predictable and well-tolerated changes, it may occasionally result in significant hemodynamic instability in susceptible patients [2].

A brief slowing of the heart rate, or transient bradycardia, often happens as a vagal response when the peritoneum is stretched as pneumoperitoneum is achieved quickly. In rare situations, this reflex can have an exaggerated response, causing marked bradycardia or even asystole. Recognizing the problem early, immediately releasing the pneumoperitoneum, and providing proper resuscitation are important for improved outcomes [3].

We present the case of a patient who developed severe bradycardia that progressed to asystole after pneumoperitoneum during an otherwise routine laparoscopic gynecologic surgery. We review some possible causes, management strategies, and prevention methods discussed in the literature. This case also highlights that certain subtle features in a patient's history might help identify those at higher risk for serious vagal reactions during laparoscopic surgery.

## Case presentation

Patient background and history

A 44-year-old woman presented for an elective minimally invasive gynecologic procedure consisting of hysteroscopy with MyoSure (Hologic, Inc., Marlborough, MA, US) hysteroscopic tissue resection, NovaSure (Hologic) radiofrequency endometrial ablation, Acessa (Hologic) radiofrequency ablation of uterine fibroids, diagnostic laparoscopy, and salpingectomy. The procedure was undertaken for the management of longstanding symptomatic uterine fibroids associated with severe dysmenorrhea and menorrhagia that were refractory to multiple conservative treatment modalities.

The patient reported a several-year history of heavy and painful menstrual bleeding, with significant worsening of symptoms over the preceding six months. Her symptoms had become increasingly disruptive to daily life, impairing both occupational functioning and caregiving responsibilities. 

Her past medical history was notable for uterine fibroids, dysmenorrhea, menorrhagia with regular cycles, and hypertension diagnosed two years ago. Although losartan 25 mg daily had been prescribed, the patient reported that she was not taking it because her home blood pressure readings were within the normal range. Her surgical history included two prior cesarean deliveries 13 and 11 years ago, with tubal ligation performed at the time of the latter procedure, as well as a prior endometrial biopsy and wisdom tooth extraction. Home medications consisted of fexofenadine 60 mg (for seasonal allergies) and a multivitamin. She reported no known drug allergies. She reported no history of tobacco use, illicit drug use, or alcohol consumption at the time of her preoperative evaluation, though later cardiology follow-up documented moderate alcohol intake (approximately five drinks per week). There was no contributory family history.

Preoperative pelvic magnetic resonance imaging demonstrated an enlarged uterus with multiple fibroids, along with mild to moderate adenomyosis and dilated pelvic vasculature. Preoperative endometrial biopsy and Pap smear were unremarkable.

On the morning of surgery, the patient was afebrile and hemodynamically stable, with a blood pressure of 127/73 millimeters of mercury (mmHg), heart rate of 76 beats per minute (bpm), respiratory rate of 12 breaths per minute, and oxygen saturation of 100% on room air.

Airway assessment revealed a Mallampati class II airway, and she was classified as American Society of Anesthesiologists (ASA) physical status II [4,5]. Physical examination was unremarkable, and a urine pregnancy test was negative.

**Figure 1:**
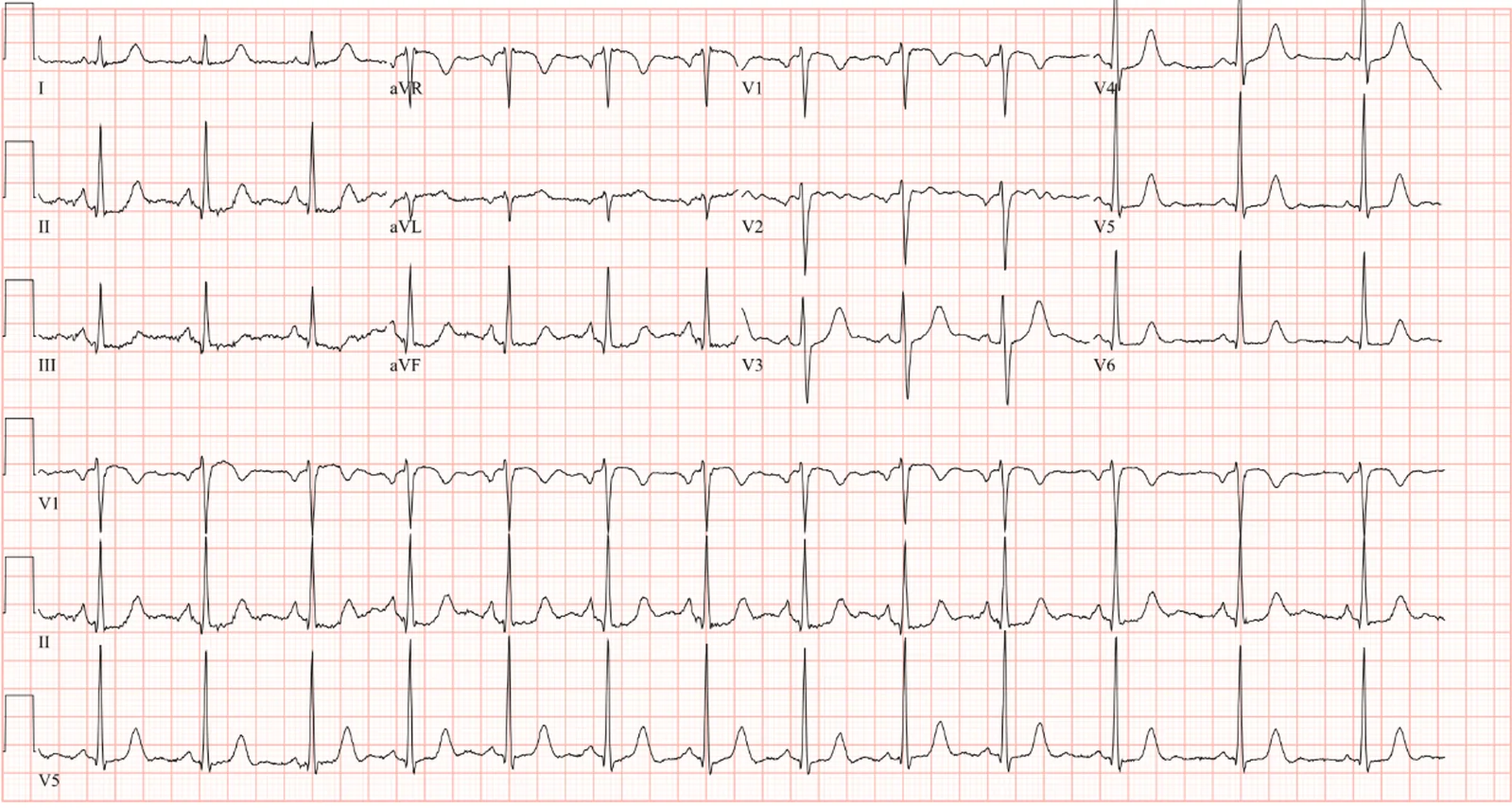
Preoperative electrocardiogram showing normal sinus rhythm without clinically significant rhythm abnormalities and a ventricular rate of 80 beats per minute

Routine preoperative laboratory studies obtained approximately two weeks prior to surgery were largely within normal limits, including complete blood count, comprehensive metabolic panel, coagulation studies, urinalysis, and hormone testing. A preoperative electrocardiogram demonstrating normal sinus rhythm without clinically significant rhythm abnormalities is shown in Figure 1.

Procedures

After informed consent was obtained, the patient was transported to the operating room and placed under general endotracheal anesthesia. Standard monitors were applied, and peripheral intravenous (IV) access was established with a 20-gauge catheter in the right forearm. Anesthesia induction consisted of intravenous midazolam 2 mg, fentanyl 100 micrograms, lidocaine 50 mg, propofol 200 mg, and rocuronium 50 mg. Endotracheal intubation was achieved on the first attempt using direct laryngoscopy with a Macintosh size 3 blade and a 7.0 millimeter (mm) cuffed endotracheal tube, without reported trauma. Tube depth was appropriate and bilateral breath sounds were heard on auscultation. Prophylactic cefazolin 2 grams (g) and dexamethasone 8 mg were administered. Mechanical ventilation was initiated in pressure-controlled volume-guaranteed mode with stable parameters and normocapnia. Sevoflurane was given for anesthesia maintenance at a concentration of 2.2%, with oxygen flowing at a rate of 2 L/min.

The intrauterine portion of the procedure was completed without complication. Examination under anesthesia revealed a retroverted uterus. Hysteroscopy demonstrated a normal endocervical canal and uterine cavity with diffuse proliferative endometrial tissue. Resection was performed using the MyoSure device. NovaSure endometrial ablation was subsequently completed in two cycles, intentionally performed by the surgeon based on their clinical experience, despite the manufacturer's recommendation for a single cycle. Repeat hysteroscopy confirmed adequate endometrial ablation, and hemostasis was achieved. A Foley catheter was placed for bladder decompression during laparoscopy. Gloves and gowns were changed in a sterile fashion, and attention was then turned to the laparoscopic portion of the procedure. After preemptive local anesthetic injection with 0.5% Marcaine with epinephrine at trocar sites, a 5 mm supraumbilical incision was made. A Veress needle was inserted, with reassuring aspiration and a successful saline drop test.

Carbon dioxide insufflation was initiated at a flow rate of 5 L/min with an opening pressure of 7 mmHg. The flow rate was subsequently increased to 40 L/min. After approximately 30 seconds and 1.3 L of carbon dioxide had been insufflated, the patient developed abrupt, profound bradycardia that rapidly progressed to asystole.

**Figure 2:**
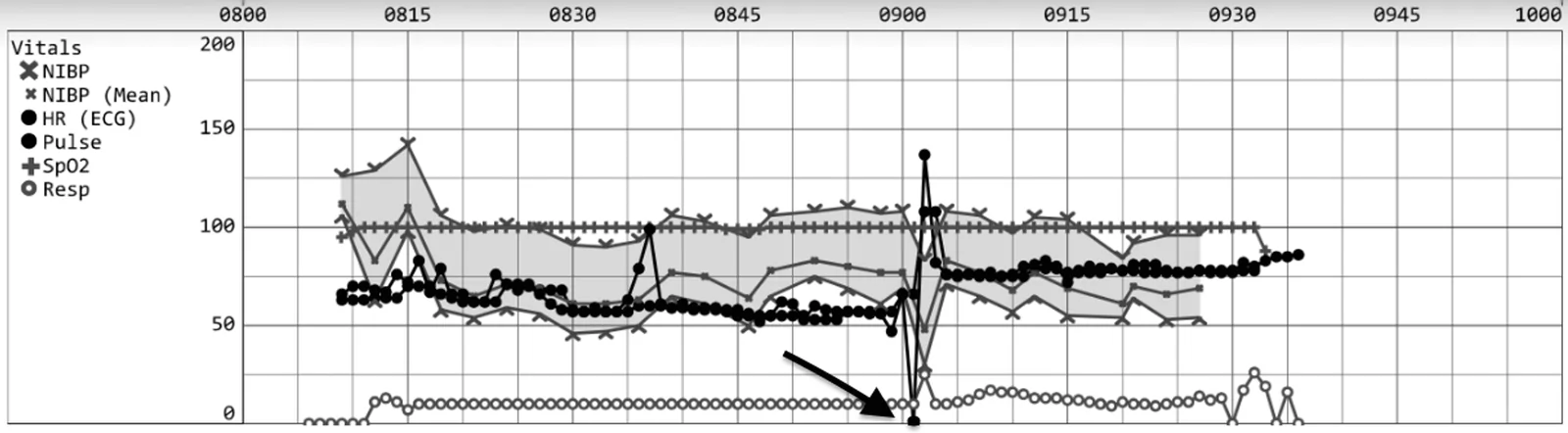
Intraoperative vital signs capturing the asystolic event marked with an arrow NIBP, noninvasive blood pressure; HR, heart rate; ECG, electrocardiogram; SpO_2_, peripheral oxygen saturation; Resp, respiratory rate

Insufflation was immediately discontinued, and the Veress needle was removed. Intravenous atropine 1 mg was administered in response to the rapidly progressive bradycardia immediately preceding the arrest. The patient subsequently became pulseless, and chest compressions were initiated. A log of intraoperative vital signs is displayed in Figure 2.

**Figure 3:**
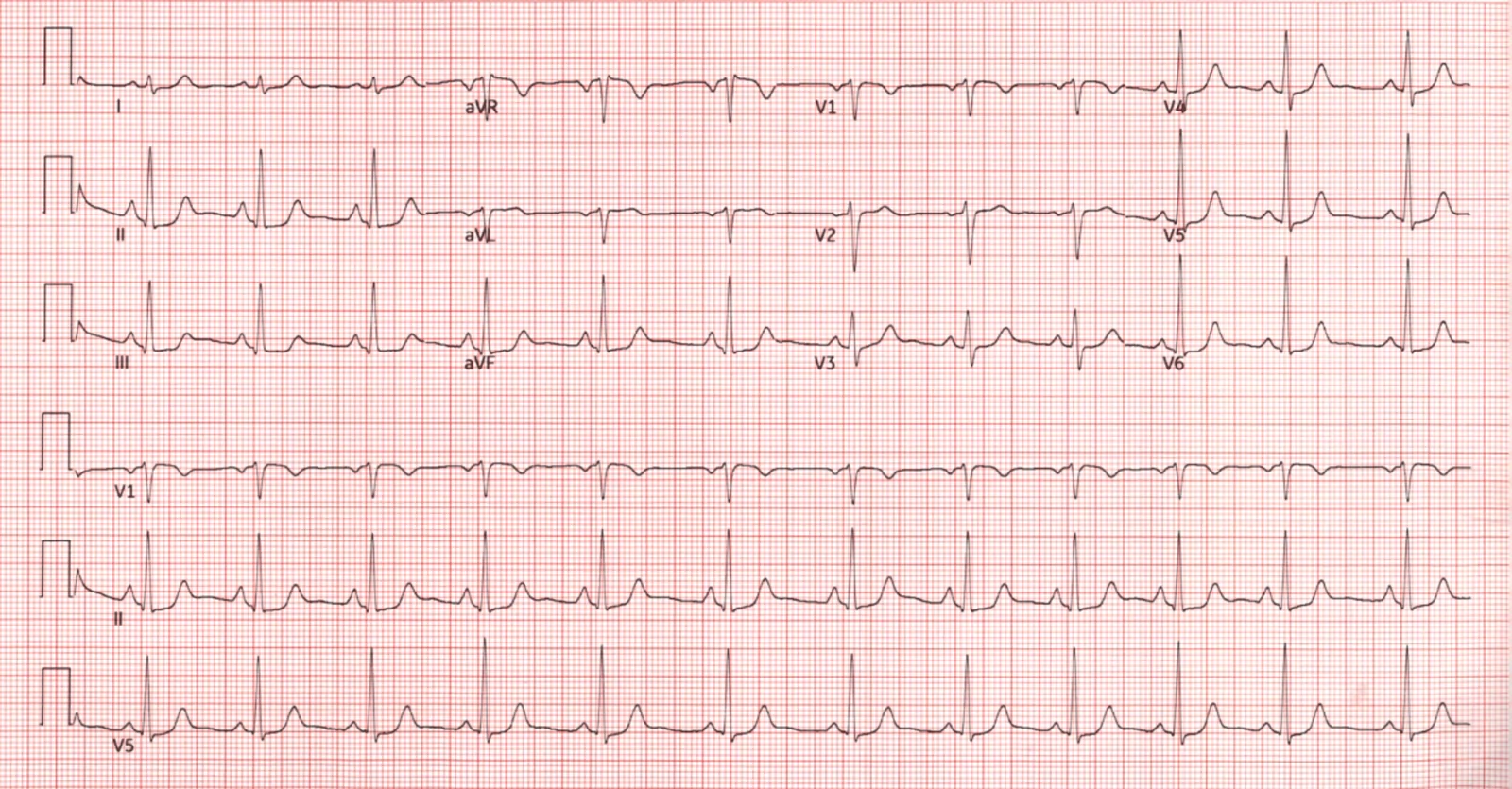
Post-return of spontaneous circulation electrocardiogram showing normal sinus rhythm with a ventricular rate of 73 beats per minute

After approximately two minutes of cardiopulmonary resuscitation, a rhythm and pulse check demonstrated return of spontaneous circulation (ROSC) with restoration of normal sinus rhythm. No epinephrine or defibrillation was administered. A bedside ultrasound was performed to ensure no obvious vascular injury or free fluid in the abdomen. Given some residual pneumoperitoneum, a Hasson entry was performed to allow for gas evacuation and direct visualization of the abdominal cavity. No vascular or bowel injury was identified. In light of the intraoperative cardiac event, the decision was made to abort the remainder of the planned laparoscopic procedure. The fascia and skin were closed in standard fashion. Neuromuscular blockade was reversed with sugammadex 200 mg, and ketorolac 30 mg was administered for postoperative analgesia prior to extubation. The patient was extubated and transferred to the post-anesthesia care unit in stable condition. The post-ROSC electrocardiogram is shown in Figure 3.

Postop

Postoperatively, the patient remained hemodynamically stable. A chest radiograph demonstrated no evidence of rib fracture or acute cardiopulmonary abnormality. Estimated blood loss was minimal, total intravenous crystalloid administration was 875 milliliters (mL), urine output was 100 mL, and endometrial curettings were sent for pathologic evaluation. The patient was noted to be alert, oriented x4, denied nausea or vomiting, and was in good spirits during the postoperative recovery period. The absence of postoperative laboratory testing represents a limitation of this report, as additional biochemical evaluation may have provided further assessment for alternative causes of the intraoperative event. However, the patient remained clinically stable postoperatively and anesthesia cleared the patient for discharge home with close follow-up recommended.

At cardiology follow-up nine days later, the patient reported a history of vasovagal-type episodes, including syncope following influenza vaccination and near-syncope during a magnetic resonance imaging (MRI) scan involving intravenous contrast administration. She also described smartwatch alerts indicating nocturnal bradycardia with heart rates in the 40-45 bpm range, resting daytime rates between 50 and 67 bpm, and appropriate chronotropic response during exercise. Blood pressure at that visit was elevated at 168/96 mmHg. Electrocardiography demonstrated a sinus rhythm with mild atrial enlargement. A transthoracic echocardiogram was performed and demonstrated a normal ejection fraction of 55-60%. Mild nonrheumatic mitral regurgitation was noted, but it was otherwise unremarkable.

## Discussion

Bradycardia leading to asystole during laparoscopy, as seen in this case report, represents a rare but potentially fatal complication during what is otherwise considered low-risk surgery. Prompt recognition and management of this complication are critical to stabilizing patients and reducing morbidities associated with prolonged hemodynamic instability [3]. This case highlights the importance of understanding the physiologic mechanisms underlying pneumoperitoneum-induced vagal reflex, recognizing effective management strategies, and identifying opportunities for prevention with thoughtful perioperative planning.

Pathophysiology

Bradycardia secondary to intra-abdominal insufflation is primarily mediated by vagal stimulation from peritoneal distention [2,3]. Secondary contributions include hemodynamic alterations from increased intra-abdominal pressure, autonomic nervous system dysregulation from vagal modulation and sympathetic attenuation from general anesthesia, and carbon dioxide absorption with hypercarbia [2].

Peritoneal distension is the dominant mechanism that leads to the activation of the vagus nerve through mechanical stretch during insufflation. This triggers a vagal reflex causing an increase in parasympathetic tone, leading to bradycardia and potentially cardiac arrest [6]. In 75% of pneumoperitoneum-related cardiac arrest cases, it is preceded by bradycardia and responds to atropine administration and pneumoperitoneum deflation, thus supporting this mechanism as the likely primary contributor to bradycardia and subsequent asystole [3].

Hemodynamic alterations also contribute. With the introduction of pneumoperitoneum, there is an increase in systemic vascular resistance and afterload while initially compressing splanchnic vasculature, thus decreasing venous return. This sudden shift leads to decreased cardiac output despite increased preload and afterload, resulting in unfavorable hemodynamic conditions [2].

Autonomic nervous system dysregulation becomes very apparent in its role, particularly when considering other surgical factors such as a steep Trendelenburg position, which increases vagal modulation [7,8]. When this occurs in conjunction with sympathetic withdrawal from general anesthesia, conditions significantly favor bradycardia [9,10]. Upon examination of heart rate variability studies, pneumoperitoneum demonstrates significant reduction of both sympathetic and vagal modulation parameters that can persist into the early postoperative period [7,11].

The insufflation medium of choice, carbon dioxide, can also contribute to significant hypercapnia via absorption and can predispose to cardiac arrhythmias. Normally, this can be managed with hyperventilation during maintenance of anesthesia and at the time of insufflation. The effects on the cardiovascular system are most pronounced at the initiation of pneumoperitoneum and correlate with duration of insufflation [3,9].

Management strategies

Immediate management of bradycardia during insufflation involves deflation of pneumoperitoneum, which will effectively reverse vagal stimulus, and administration of atropine [2,6]. Per the American Heart Association Advanced Life Support guidelines for symptomatic bradycardia, 0.5-1.0 mg IV of atropine every 3-5 minutes, up to 3.0 mg, is recommended. For refractory bradycardia causing hemodynamic instability that is not controlled with medication, transcutaneous pacing should be initiated [12]. Second-line options include epinephrine infusion (2-10 mcg/min) or dopamine infusion (5-20 mcg/kg/min), both of which increase heart rate through beta-adrenergic stimulation [12]. 

If progression to asystole occurs, immediate cardiopulmonary resuscitative efforts must be initiated following Advanced Cardiac Life Support (ACLS) guidelines. As IV access is already in place, this should occur in conjunction with continued medication management [12].

Preventative measures: laparoscopic preoperative planning

In high-risk patients, prophylactic placement of transcutaneous pads is reasonable. High-risk patient populations include those >60-65 years old, ASA class III-IV, baseline heart rate <60 bpm, or those who are taking beta-blockers or calcium channel blockers [12,13].

The use of prophylactic anticholinergic agents is controversial. A systematic review found insufficient evidence supporting the routine use during gynecological surgery, but several small studies evaluated the effect of anticholinergics in the setting of less commonly used regimens for anesthesia induction such as fentanyl, halothane, and vecuronium. These studies showed statistically decreased bradycardia, but halothane is no longer a common agent for induction of anesthesia due to its cardiovascular and hepatic toxicity [14-16].

Intraoperative methods of prevention

The most extensively studied intervention is low-pressure pneumoperitoneum, which consists of using insufflation at a pressure of 8-10 mmHg rather than the standard 12-15 mmHg. By reducing pneumoperitoneum, peritoneal stretch is reduced, as is carbon dioxide absorption. In patients with cardiac dysfunction, ASA III-IV specifically, reducing insufflation pressure is critical if invasive methods are used for arterial pressure monitoring [17].

Gradual insufflation is practical and often overlooked as a strategy to decrease the incidence of bradycardia. Surgeons tend to use low flow (1-5 L/min) as opening pressure is being established to confirm intraperitoneal placement and proceed to increase to high flow (20-40 L/min) [3,17]. Insufflating an abdomen at low flow versus high flow increases operative time minimally from a clinical standpoint. A typical volume requirement of 3.5-5L within the abdomen and reaching 12-15 mmHg for working conditions, low flow requires approximately 40-60 seconds vs 10-15 seconds at high flow. This avoids rapid peritoneal distention, which is one of the key mechanisms that causes the abrupt vagal reflex leading to bradycardia [17].

Alternate anesthetic strategies include avoidance of excessive sympatholytic agents and use of low-dose ketamine adjunct [17,18]. Ketamine administration at 0.5 milligrams per kilogram (mg/kg) in addition to common anesthesia regimens reduced the incidence of decreased heart rate from 53% to 10% [18].

Clinical implications

This case suggests that certain patients may have a predisposition to experiencing a severe vagal reflex upon initiating pneumoperitoneum that is otherwise not identified during a standard preoperative assessment. After the event, our patient described a history of vasovagal reactions after vaccination and during an MRI, with intermittently low resting heart rates, possibly indicating an increased vagal tone at baseline. Although these observations cannot establish causation, we hypothesize that seemingly benign historical features may identify an increased risk for dangerous vagally mediated cardiovascular events during establishment of pneumoperitoneum. This hypothesis is supported indirectly by prior studies demonstrating that a recent history of vasovagal syncope is strongly associated with recurrent vasovagal episodes [19]. Whether this association extends to vagally mediated intraoperative cardiovascular events remains unknown. Accordingly, the available evidence is insufficient to support a formal screening protocol or the routine use of prophylactic medications. However, obtaining a focused history regarding vasovagal syncope, persistent bradycardia, and previous exaggerated vagal episodes is a low-cost and quick intervention that may improve perioperative awareness of a potential issue. Future studies are needed to determine if these historical features can accurately predict vasovagal reactions or related cardiovascular events during pneumoperitoneum.

## Conclusions

This case likely describes a rare instance of vagally mediated asystole following carbon dioxide insufflation of the abdomen during a routine laparoscopic gynecologic surgery. Immediate evacuation of the pneumoperitoneum, administration of atropine, and initiation of cardiopulmonary resuscitation led to a rapid recovery of normal circulation with no obvious aftereffects. While this complication is rare, it requires a high degree of vigilance for severe vagal reactions during abdominal insufflation.

The patient's previous history of vasovagal episodes suggests that some aspects of a patient’s history might help identify individuals who are more likely to have excessive vagal responses during pneumoperitoneum. Although there is not yet enough evidence to recommend formal screening or preventive measures based only on these findings, asking about past vasovagal episodes and baseline bradycardia before surgery is easy, economical, and might highlight at-risk patients during the perioperative period. Additional research is needed to determine whether these historical features reliably predict serious vagally mediated cardiovascular events during surgery.
